# Head-neck rotational movements using DidRen laser test indicate children and seniors’ lower performance

**DOI:** 10.1371/journal.pone.0219515

**Published:** 2019-07-25

**Authors:** Renaud Hage, Fabien Buisseret, Laurent Pitance, Jean-Michel Brismée, Christine Detrembleur, Frédéric Dierick

**Affiliations:** 1 Laboratoire NMSK, Institut de Recherche Expérimentale et Clinique, Université catholique de Louvain, Brussels, Belgium; 2 Forme & Fonctionnement Humain Lab, CeREF, Haute Ecole Louvain en Hainaut, Charleroi, Belgium; 3 Stomatologie et Chirurgie Maxillo-Faciale, Cliniques Universitaires Saint-Luc, Brussels, Belgium; 4 Center for Rehabilitation Research, Texas Tech University Health Sciences Center, Lubbock, Texas, United States of America; University of Innsbruck, AUSTRIA

## Abstract

Sensorimotor control strategies during cervical axial rotation movements have been previously explored in narrow age ranges but never concurrently in Children and Seniors during a well-standardized task. However, the lifespan developmental approach provides a framework for research in human sensorimotor control of the head-neck complex. A cross-sectional design was used to investigate the influence of age on head-neck dynamic performance adopted by asymptomatic Children, Adults and Seniors using a standardized task (DidRen Laser test). Participants performed 5 cycles of left/right head-neck complex fast rotational movements toward 3 targets with 30° of angular separation. Dynamic performances were computed from total execution time of the test and kinematic variables derived from rotational motion of head measured by an optoelectronic system. Eighty-one participants, aged 8–85 yrs, were stratified in four groups: Children, Younger adults, Older adults and Seniors. Children were significantly slower than Younger (p<0.001) and Older adults (p<0.004) and Seniors slower than Younger adults (p<0.017) to perform the test. Children adopted a lower average speed compared to Younger (p<0.001) and Older adults (p<0.008). Children reached the peaks speed significantly later than Younger (p<0.004) and Older adults (p<0.04) and acceleration significantly later than Younger (p<0.001) and Older adults (p<0.013). From the peak acceleration, Children reached end of the cycle significantly slower than Younger (p<0.008) and Older adults (p<0.008). Children significantly differed from all other groups for rotational kinetic energy, with smaller values compared to Younger adults (p<0.001), Older adults (p<0.005) and Seniors (p<0.012). Variability was also significantly higher for Seniors and Children. In conclusion, age influences head-neck visually elicited rotational dynamics, especially in Children. These results suggest that age should be taken into account when establishing normative data and assessing dynamic head-neck sensorimotor control of patients with neck pain.

## Introduction

The perspective of life span development provides a framework to study how and why individual cognitive and sensorimotor control changes occur across the whole lifespan [1–3]. Pioneering research studies showed a close relationship between cognitive tasks and performance [2]. More recently, mirroring results were observed for several motor tasks with (inverted) U-shaped relationships between performance and age [4]. From a structural point of view, changes in the brain were previously observed with U-shaped curves with low white matter volume in both children and old adults [4–7]. These results may be explained by Neural Darwinism [8]. This theory of a general model of lifespan process of nervous system development is built on the principle that the development is based on a process of selection that takes place inside the nervous system [8, 9]. The connections that are used the most become stronger, while the others disappear, thus creating neural networks that are unique to each individual. It is through this process that motor behaviors come into being [8, 9].

Most developmental research has either focused on changes in early development or on aging, and knowledge about the general principles of life long development is nowadays still considered as insufficient [4]. It is known that sensorimotor control decreases in old age due to deterioration of vestibular, visual and neuromuscular functions [10, 11]. This deterioration can be highlighted by reduction of performance in various motor tasks such as gait, fine motor hand and even cervical fast neck movements [12–16]. Sensorimotor control could also be lowered by immaturity of the central, peripheral nervous and musculoskeletal systems’ immaturity in healthy children [17, 18]. Indeed, existing research indicates that motor performance improves from childhood to adolescence [17] and early development is characterized by an increase in performance, such as decrease in reaction time, and an increase in processing speed and intelligence [4].

Previous studies explored postural [19], locomotor [20] and grasping control development [13, 21] with, for example, hand-eye coordination task [4] across the lifespan. However, visually elicited head-neck complex rotational motor control across lifespan has been insufficiently explored despite the major importance of their reliance on afferent information from vestibular, visual and proprioceptive systems which converge in several areas throughout the central nervous system [22] and the existence of various models of head-neck complex kinematics in the horizontal plane [23–26].

To our knowledge, only the study of Bahat et al. (2016) explored the kinematics of the head and the symmetry of velocity profile in response to virtual targets appearing randomly in subjects aged 18 to 80 years [16]. This study showed that age influences angular speed, specifically in elders over 60 years. However, it is not possible to generalize their results to a real environment, to movements mainly executed in a specific plane, and to other ages such as in children.

In this context of studying the sensorimotor control system performance of the head-neck complex dynamics, the DidRen Laser test was developed [27]. It consists of a standardized test during which the participants must realize fast, low amplitude, and accurate axial rotational movements of the head in response to real visual targets that must be hit by a laser beam placed on the participants’ head. Indeed, this test is particularly useful by offering the advantage to focus on the neck sensory and motor control systems with many direct neurophysiological connections between the proprioceptive, visual and vestibular systems [28]. Firstly, it emphasizes real visual targets completed by an auditory feedback system. When the laser beam is pointed correctly at the target, the sensor lights up and the system emits an audible sound signal. Secondly, with the fast and accurate head rotation movement of approximately 30°, the head-neck complex remains within an amplitude range, without strain for the passive system (joint capsules and facets and intervertebral disks and ligaments). In contrast, the head-neck complex intensively draws on the active system, i.e. on the spinal muscles providing dynamic stability [29] while also stimulating the vestibular system [30]. Today, there is a need for more research examining how neck rotation dynamics could be influenced by age and specifically the realization of normative data during a standardized task such as the DidRen Laser test that maximizes input from the sensorimotor control system [27–30].

The objective of this cross-sectional study was to investigate the influence of age from childhood to elderly on head-neck dynamic performance using the DidRen Laser test. Performance and speed profile used by the participants were assessed by computation of total execution time and by exploring kinematic variables derived from head-neck complex rotational movements.

## Material and methods

### Participants

Eighty-one children and adults (43 females, 38 males) recruited consecutively from a sample of convenience from colleagues in University hospital and among researchers’ acquaintances volunteered to participate in this study. They were stratified by age in four groups: Children [Ch, 8–14 yrs, n = 17]; Younger adults [YA, 18-35yrs, n = 29]; Older adults [OA, 36–64 yrs, n = 18]; and Seniors [S, 65–85 yrs, n = 17]. To ensure complete maturation of the sensorimotor representation, children-age was limited to 14 years [18]. Moreover, we selected to split adults participants into two groups because neck pain prevalence increases with age and is most common around the fifth decade of life [31].

The participants or children legal representatives signed informed consent. The individual in this manuscript has given written informed consent (as outlined in PLOS consent form) to publish these case details. The study was approved by the local ethics committee (Comité d’Ethique Hospitalo-Facultaire Saint-Luc-UCL (IRB 00001530)) and conducted in accordance with the declaration of Helsinki.

Participants did not exhibit any neuromusculoskeletal or neurologic disorder that could influence the performance of head-neck complex rotation in the horizontal plane. Inclusion criteria were the absence of neck pain episodes in the last 6 months and a Neck Disability Index (NDI) [32] score of less than or equal to 4% [33]. Exclusion criteria were: impaired cognition, blindness, deafness, dizziness or vestibular disorders diagnosed by a physician. Visual Analogue Scale (VAS) was also used to confirm the absence of neck pain on the testing day [34, 35].

### Instrumentation

**Fig 1:**
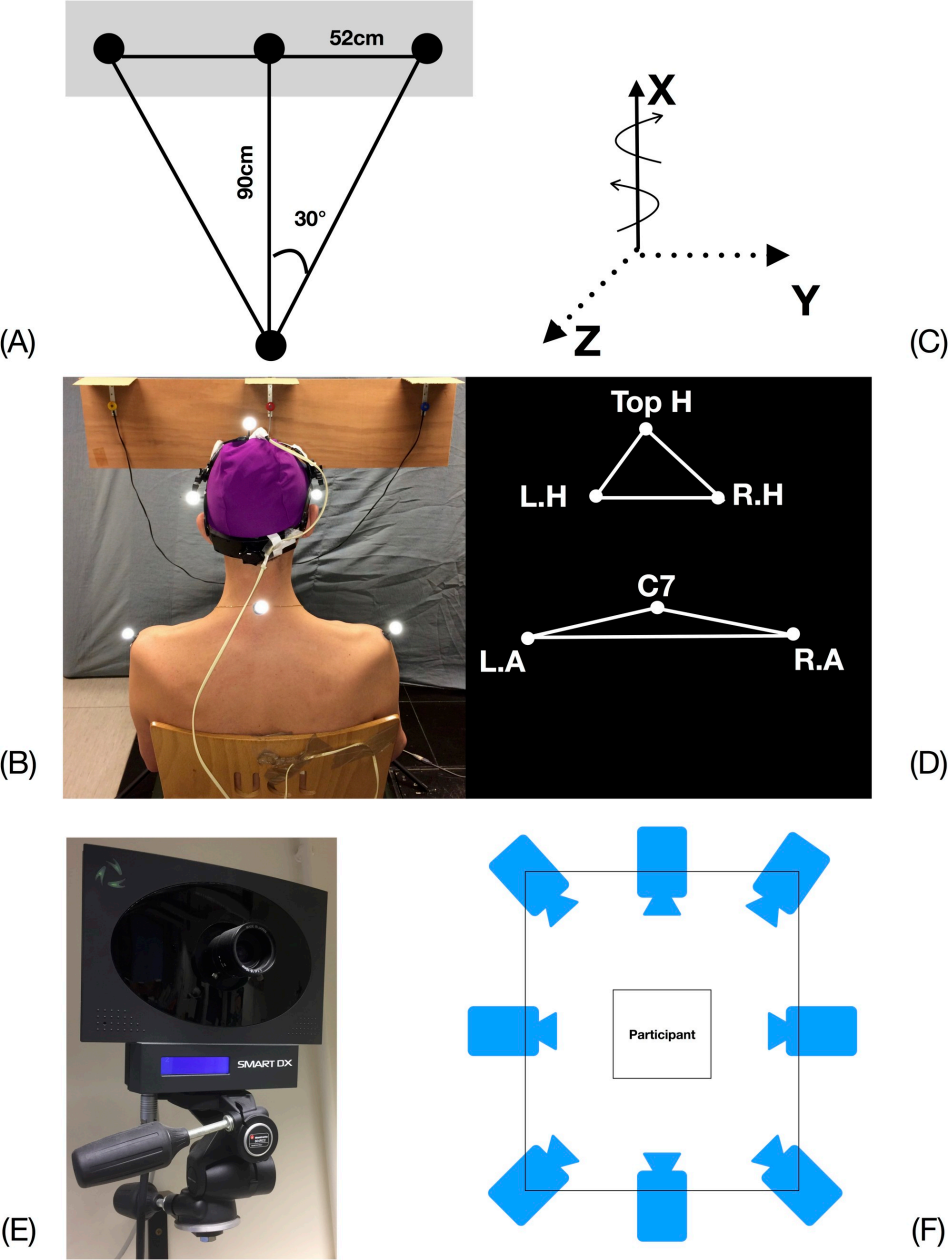
DidRen Laser, kinematic model and instrumentation. (A) DidRen Laser installation device including the 3 photosensitive sensors. (B) The placement of markers on the helmet (Head = H, Left Head = L.H, Right Head = R.H) and the shoulders (Left Acromion = L.A, Right Acromion = R.A, 7^th^ Cervical vertebrae = C7). (C) Head rotation is executed around a vertical axis (X) in a horizontal plane (Y-Z). (D) Segments modeled as two triangles with reflective markers on the head and the trunk. (E) One infra-red camera. (F) Representation of the field of vision of the infra-red cameras.

The DidRen Laser test was used to standardize the visually elicited rotational motion of the participant’s head-neck complex. A detailed description of the system and its components has been previously published and is summarized in Fig 1A [27]. It is composed of three photosensitive sensors including photocells, a plastic adjustable helmet to which a fine laser beam is attached and worn by the subject, and a computer equipped with a customized software that calculates the time between two consecutive “hits” of the sensors. A chair without armrests was placed at 90 cm from a vertical panel equipped with the 3 sensors arranged horizontally and located 52 cm apart (Fig 1A). While the participant fixed her/his gaze on the central sensor, the experimenter adjusted the laser beam onto the target. This procedure allowed to ensure the participant kept her/his head in a neutral position before beginning the test. The distance between the participant and the wall was chosen so that the participant performed a 30° rotation both to the left and right sides of the bodyline. Little rotation amplitude was chosen to remain within an amplitude range, which would not place joints capsules, facets and intervertebral disks and ligaments under strain, while maximizing input from the sensorimotor control system [27].

Three-dimensional markers position recording was completed at a sampling frequency of 200 Hz during the DidRen Laser test [27], using an optoelectronic system with 8 infra-red cameras (ELITE-BTS, Milan, Italy) (Fig 1E). A kinematic model composed of 2 x 3 markers on a helmet and fixed during all experimentations (Fig 1B), adapted from Bulgheroni et al. (1998), representing the head and trunk segments was used. Head segment was modeled as a first triangle with three reflective markers fixed to a helmet worn on the head by the participant (Fig 1B and 1D). Helmet markers were positioned such that one coincided with top of the vertex (Top. H), and two on each sides of the top (R. H and L. H). Trunk segment was modeled as a second triangle with left/right acromioclavicular joints (L. A and R. A) and the spinous process of C7 vertebra (Fig 1B and 1D)[36]. Real time detection of head rotation markers was executed around a vertical axis (X) in a horizontal plane (Y-Z). The movement occurred around the global coordinate system. The system was preliminary calibrated inside the field of vision of the infra-red cameras [37] (Fig 1F).

### Experimental procedure

The participants watched an explanatory video of the experimental procedure before the first measurements. The participants sat on a chair in a comfortable position, the back against the backrest, the palm of hands on the thighs, the soles of the feet on the floor with heels against a stop block placed at the feet of the chair. While the participant fixed his gaze on the central target (previously placed at eye-level using a magnetic media), the experimenter adjusts the helmet with the laser beam onto the target. The participant then confirmed that the laser beam was indeed pointing at the spot at which he/she was looking. Instructions for the execution of the test were clear for all participants: “you must reach the targets as fast as you can”. Participants were also instructed to perform only a fast rotation of the head-neck complex without rotating the trunk. This imply that the participants had to trade-off the speed phase (during head rotation) and the accuracy phase (during stabilization of the head).

Analysis was done on head-neck complex rotations. Trunk rotations were only recorded to verify that trunk motion in the horizontal plane was negligible.

**Fig 2:**
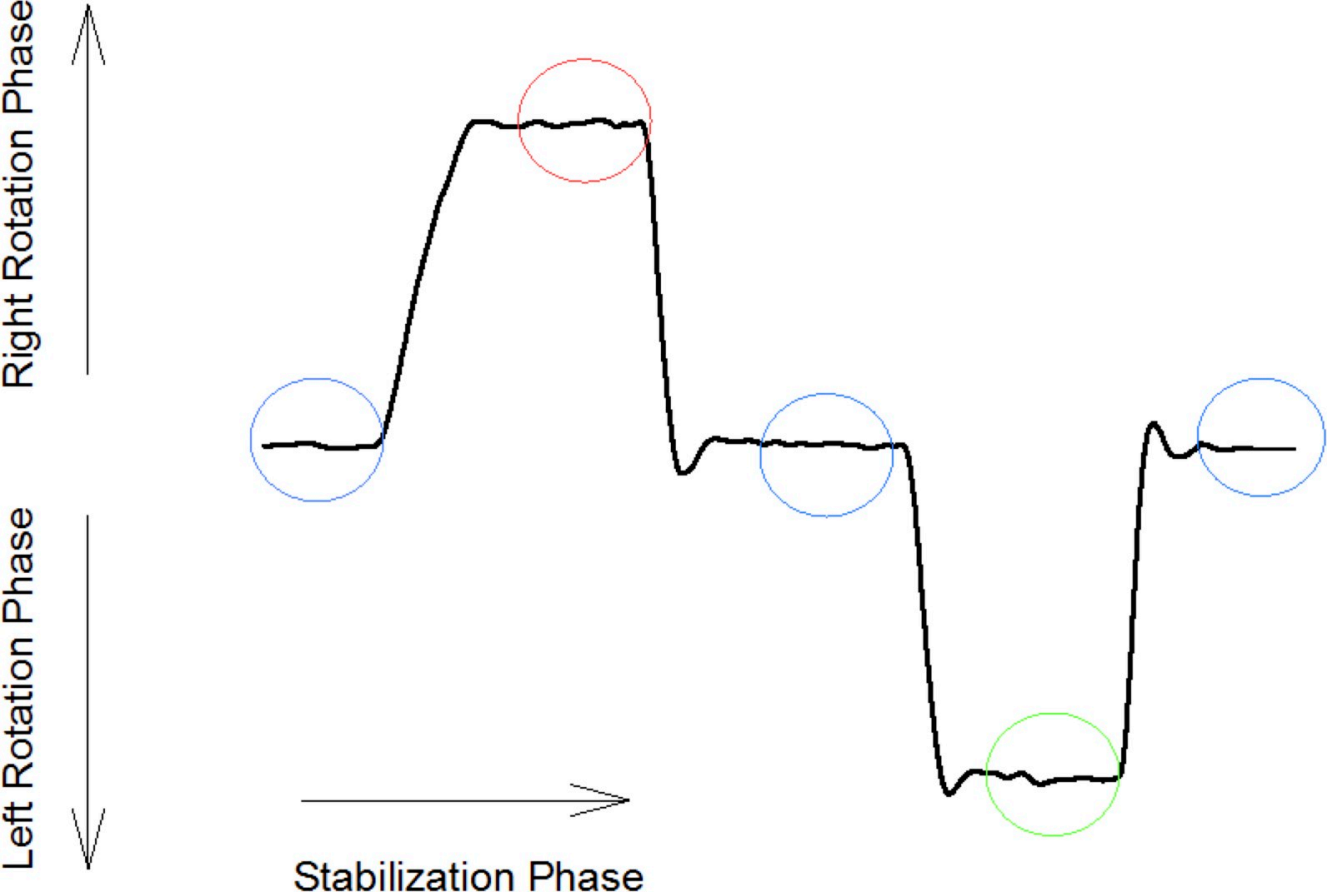
Typical trace recorded during head rotations. Head rotations phases are visualized by the ascendant/descendant phase of the solid line and stabilization phases are visualized by the horizontal phase of the solid line. Blue circles are for center target. Red circle is for right target. Green circle is for left target.

Two DidRen Laser tests were conducted. The procedure was as follow: when the laser beam was on the target/sensor (during at least 0.5 s), the LED’s sensor lighted up and the system emitted a sound signal. A complete trial was composed of 5 cycles with 5 rotations to the right and 5 rotations to the left. As soon as the central sensor ‘‘hit”, the participants were asked to turn the head to the right to hit the right-hand sensor as quickly as possible. Then they returned to the central sensor and then moved on to hit the left-hand sensor. The participants completed the cycle by hitting the central sensor for a third time. Therefore, left and right rotation toward the target was composed of two phases. One fast rotation phase to turn the head followed by one stabilization phase to adjust the laser accurately during 0.5 second in the sensor/target (Fig 2). The first trial was used to “familiarize” the participant with the experiment and to minimize a training effect, and the second trial for data collection and analyses.

### Measured and computed kinematic variables

From each instantaneous X, Y, and Z coordinate, head-neck complex and trunk angular displacement was computed using homemade software.

**Fig 3:**
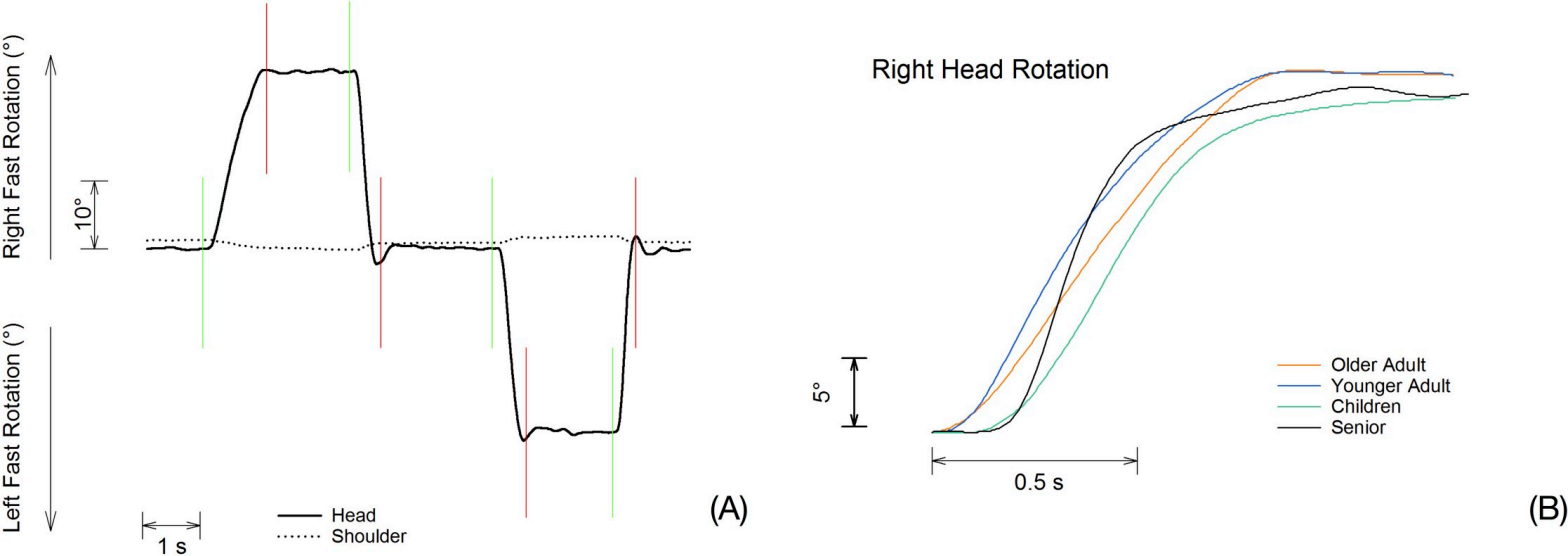
Typical trace during one complete cycle and example of different performances achieved by individuals of different age-group. (A) Typical trace of head-neck complex and trunk rotation during one complete cycle (center-right-center-left-center). Analysis was conducted during head rotation (ascendant/descendant solid line) phase with placement of cursors from the beginning (green) to the end of the rotation (red). (B) Example of right axial rotation performed by individuals of different age-group.

By numeric finite difference, we calculated head-neck complex angular speed and acceleration from beginning to the end of each rotation cycle (Fig 3). Angular displacement was calculated by software as described in detail by Davis et al.[38].

To calculate the speed (*ω*) at frame i, we used the formula: $\omega_{i}=\frac{\theta_{i+n}-\theta_{i-n}}{2\;.n.\Delta t}$ where *θ* = angular displacement; n = 5 and Δ*t* = 1/sampling frequency. To calculate the acceleration (α) at frame i, we used the formula: $\alpha_{i}=\frac{\omega_{i+n}-\omega_{i-n}}{2\;.n.\Delta t}$, where *ω* = angular speed; n = 5 and Δ*t* = 1/sampling frequency.

**Fig 4:**
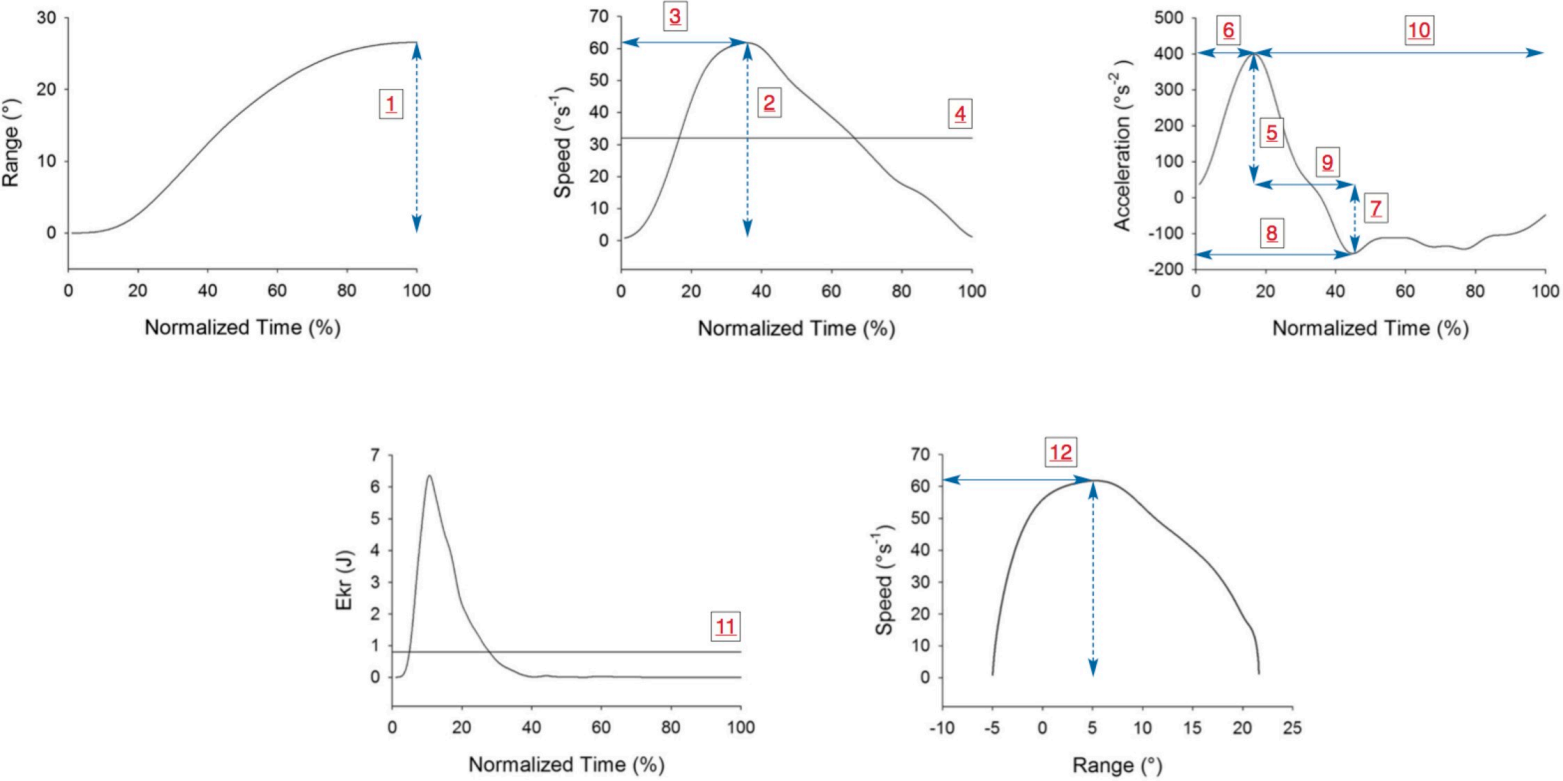
Typical plots of variables analyzed during one right rotation in a participant of the Younger adult’s group (age: 22 yrs, sex: female). All values are in absolute value. (1) range of motion (°); (2) peak speed (° s^-1^); (3) time to peak speed (s); (4) average speed (° s^-1^); (5) peak acceleration (° s^-2^); (6) time to peak acceleration (time to achieve the peak of acceleration (s); (7) peak deceleration (° s^-2^); (8) time to peak deceleration (s); (9) time between peaks acceleration and deceleration (s); (10) time from peak acceleration to end of rotation (s); (11) Average rotational kinetic energy (J); (12) value of the angle when maximum speed is achieved (°).

The following specific parameters were computed for each angular variable (Fig 4): (1) range of motion (ROM, in °); (2) maximum angular speed achieved (peak speed, in °s^-1^); (3) time to reach the peak speed (time to peak speed, in s); (4) average speed (in °s^-1^); (5) maximum angular acceleration reached (peak acceleration, in °s^-2^); (6) time to reach peak acceleration (time to peak acceleration, in s); (7) minimum of angular acceleration (peak deceleration, in °s^-2^); (8) time to reach peak deceleration (time to peak deceleration, in s); (9) time between maximum angular acceleration reached and minimum of angular acceleration (time between peaks of acceleration and deceleration, in s); (10) time from peak acceleration to end of cycle (in s); and (11) head moment of inertia (*I*), which was estimated based on a regression equation [39] using anthropometric measurements of Rollins et al. [40]. The formula used was: *I* = 0.223*P*^5^ with *P* = head perimeter in *m* and gives *I* in *kg m*^2^.

The knowledge of *(I)* allowed the computation of the instantaneous rotational kinetic energy (in J) of each participant’s head, which was subsequently calculated using head’s instantaneous angular speed (*ω*), according to the following equation: *Iω*^2^/2; (12) value of the angle-speed phase plane (angle at maximum speed, in °).

All variables were calculated during 5 consecutive cycles and averaged.

The DidRen homemade software calculates the time taken by the participant to go from one ‘‘hit” sensor to another and the DidRen total time (in s) to complete the 5 cycles of one trial. The DidRen total time includes the fast rotation and the stabilization phases (from the first to the last target).

### Statistical analyses

**Fig 5:**
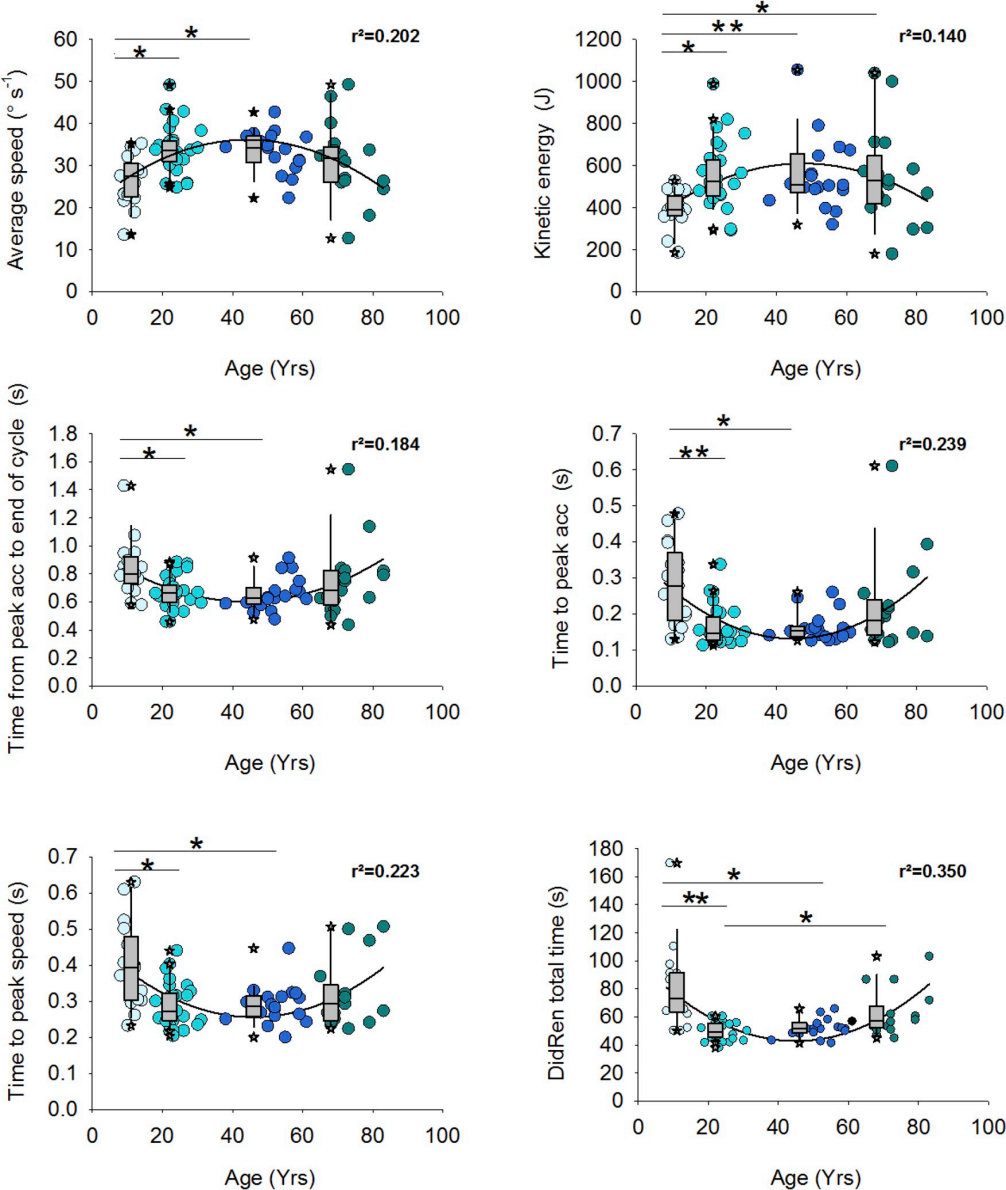
Box-plots and individual results for significant variables according to the different age-groups. Temporal performance (DidRen total time) and head-neck complex dynamics are represented as function of age and significant post hoc comparisons: * p<0.05; ** p<0.001. Second order polynomial regression curves (solid lines) were fitted across all data using the method of least squares.

The coefficient of variation ($CV=\frac{SD}{MEAN}$, in %) was computed for all variables to assess data spread around the average value CV for each variable, except for DidRen total time, was computed for each participant on the five cycles. Effect of age on the variables and their CV was assessed by a one-way Analysis of variance (ANOVA) with *post hoc* Holm-Sidak method for pairwise multiple comparisons when normally distributed (tested by Shapiro-Wilk) and one-way ANOVA on ranks with *post hoc* Dunn's method for pairwise multiple comparisons if normality test failed. All statistical procedures were performed with SigmaPlot 13 (Systat Software, Inc.). Significance was determined at p<0.05. Second order polynomial regression curves (solid lines) were fitted across all data using the method of least squares (Fig 5). All Figures show a U-shaped or inverted U-shaped age profile that especially emphasizes differences related to aging.

Average of the data recorded from the trunk rotations was calculated using the data of five participants randomly selected from each age-group.

## Results

A total of 88 participants were recruited with 7 excluded due to an NDI score >4%. The main characteristics of the participants are listed in Table 1.

**Table 1:** Characteristics of the participants according to the four age groups.

Participants	Global (n = 81)	Children (n = 17)	Younger adults (n = 29)	Older adults (n = 18)	Seniors (n = 17)
Age (years), mean±SD	37.6±23.4	11±1.8	24±3	53±7	73±5
Sex *(males/females)*, n	39/42	6/11	13/16	11/7	9/8
BMI (*kg m*^*-*^*²)*, mean±SD	22.3±4.3	16.9±2.1	22.9±3.0	24.3±4.2	24.5±3.2
NDI (100), median [Q1-Q3]	2 [0–4]	0 [0–2]	2 [1–2]	2 [1–4]	2 [0–4]
VAS (0–10), median [Q1-Q3)	0 [0–0]	0 [0–0]	0 [0–0]	0 [0–0]	0 [0–0.5]

Global = all age-groups, SD = Standard Deviation, BMI = Body Mass Index, Q1 = First Quartile, Q3 = Third Quartile, NDI = Neck Disability Index

The results for all variables collected during the DidRen laser test are described in Table 2.

**Table 2:** Results for variables collected during the DidRen test according to the four age groups.

Variables	Tests	Ch	YA	OA	S	Comparisons between groups (Power)
ROM (°)	Mean±SD	25.9 ± 1.4	26.1 ± 0.8	25.5 ± 1.4	25.5 ± 1.1	# (0.09)
	CV Median [Q1-Q3]	3.5 [2.0–4.7]	3.1 [2.0–4.5]	3.0 [2.1–4.2]	3.1 [2.3–4.8]	# (0.05)
Peak speed (°s^-1^)	Median [Q1-Q3]	74.4 [71.5–81.9]	79.8 [70.0–96.3]	77.6 [69.0–92.1]	85.8 [64.1–95.6]	# (0.05)
	CV Median [Q1-Q3]	23.4 [15.3–24.7]	14.6[12.2–17.9]	15.9 [11.8–19.4]	15.8 [12.6–24.8]	Ch>YA* (0.64)
Time to peak speed (s)	Median [Q1-Q3]	0.39 [0.30–0.5]	0.27 [024–0.32]	0.29 [0.26–0.32]	0.29 [0.25–0.34]	Ch > YA*, OA* (0.96)
	CV Median [Q1-Q3]	25.5 [18.5–35.7]	18.9 [15.5–25.3]	22.2 [12.9–25.7]	22.4 [16.0–30.4]	# (0.61)
Average speed (°s^-1^)	Mean±SD	26.4 ± 5.8	33.9 ± 5.5	33.5 ± 4.8	31.0 ± 9.0	Ch > YA*, OA* (0.88)
	CV Mean±SD	21.9± 4.9	17.1± 4.1	18.5± 6.9	20.2± 4.9	Ch>YA* (0.62)
Peak acceleration (°s^-2^)	Median [Q1-Q3]	542.0 [512.0–615.7]	592.5 [474.8–704.3]	534.6 [442.5–733.9]	661.7 [394.6–74.2]	# (0.05)
	CV Median [Q1-Q3]	26.5 [19.5–39.2]	21.9 [18.7–26.1]	19.9 [17.8–30.3]	23.8 [19.5–39.2]	# (0.33)
Time to peak acceleration (s)	Median [Q1-Q3]	0.28 [0.18–0.37]	0.15 [0.13–0.19]	0.15 [0.14–0.16]	0.18 [0.14–0.24]	Ch > YA**, OA* (0.98)
	CV Median [Q1-Q3]	33.6 [26.5–46.7]	29.6 [21.1–33.9]	32.3 [25.1–40.1]	30.8 [20.4–35.8]	# (0.52)
Peak deceleration (°s^-2^)	Median [Q1-Q3]	-366.3 [-426.7–297.8]	-364.8 [-510.9–292.2]	-377.3 [-462.1–282.0]	-433.2 [-529.2–-291.2]	# (0.05)
	CV Median [Q1-Q3]	-38.1 [-46.4–30.4]	-30.3 [-33.4–25.1]	-28.3 [-34.1–25.6]	-30.3 [-39.7–23.9]	# (0.69)
Time to peak deceleration (s)	Median [Q1-Q3]	0.56 [0.42–0.65]	0.45 [0.36–0.50]	0.41 [0.39–0.49]	0.41 [0.36–0.61]	# (0.54)
	CV Median [Q1-Q3]	30.5 [21.7–43.4]	26.1 [20.1–28.3]	28.2 [18.6–30.9]	27.0 [17.3–31.4]	# (0.49)
Time between peaks acceleration-deceleration (s)	Median [Q1-Q3]	0.26 [0.24–0.33]	0.27 [0.23–0.23]	0.26 [0.22–0.34]	0.25 [0.24–0.33]	# (0.05)
	CV Mean [Q1-Q3]	55.7 [44.0–81.2]	39.1 [31.7–43.9]	35.6 [26.7–50.8]	37.4 [24.3–55.7]	Ch>OA*, YA*, S* (0.85)
Time from peak acceleration to end of cycle (s)	Mean [Q1-Q3]	0.80 [0.73–0.92]	0.66 [0.60–0.72]	0.63 [0.59–0.70]	0.68 [0.58–0.82]	Ch >YA*, OA* (0.77)
	CV Mean [Q1-Q3]	27.4 [24.9–32.4]	21.6 [18.3–25.6]	21.2[18.4–26.1]	23.7 [20.3–27.4]	Ch>YA*, OA* (0.70)
Kinetic energy (J)	Median [Q1-Q3]	389.5 [363.2–453.8]	524.2 [453.9–629.3]	507.3 [472.3–654.2]	530.7 [417.3–646.8]	Ch < YA**, OA*, S* (0.75)
	CV Median [Q1-Q3]	24.8 [15.9–31.3]	19.1[15.8–23.8]	17.6 [14.2–23.7]	20.1 [16.7–28.5]	# (0.51)
Angle at maximum speed (°)	Median [Q1-Q3]	13.9 [12.7–14.6]	12.9 [12.1–13.7]	12.5[11.9–13.1]	12.9 [12.2–14.1]	# (0.09)
	CV Mean±SD	63.9± 12.2	49.3±12.4	48.8±12.3	60.4±12.2	S>YA*, OA*; Ch>YA**, OA** (0.98)
DidRen total time (s)	Median [Q1-Q3]	69.7 [57.4–89.4]	49.6 [45.6–55.6]	51.7 [48.4–55.8]	57 [52.3–67.6]	Ch> YA**, OA* S > YA* (0.98)

For all variables, if normality test passed results were in mean± Standard Deviation (SD) and if normality test failed results were in median with interquartile range [Q1-Q3]. CV = coefficient of variation in % and average. Ch = Children, YA = Younger adults, OA = Older adults, S = Seniors. No age-group significant difference = (#). Age-related significant difference is observed for 4 kinematic variables, for time duration ((DidRen total time) and for Kinetic energy. Indications for which group differed from another: Longer or Slower = (>), Less = (<).

* p<0.05;

** p<0.001. Values between parentheses are for statistical power of the tests.

Children were significantly slower than Younger (p<0.001) and Older adults (p<0.004) and Seniors slower than Younger adults (p<0.017) to perform the test. No significant differences between Younger and Older adults were found for all kinematic variables. Children reached the peaks speed significantly later than Younger (p<0.004) and Older adults (p<0.04) and acceleration significantly later than Younger (p<0.001) and Older adults (p<0.013). Children significantly differed from all other groups for average kinetic energy, with smaller values compared to Younger adults (p<0.001), Older adults (p<0.005) and Seniors (p<0.012). Children showed significant slower Average speed compared to Younger (p<0.001) and Older adults (p<0.008). From the peak acceleration, Children reached end of the cycle significantly slower than Younger (p<0.008) and Older adults (p<0.008). Regression Parabolic regression curves graphically display the U-shaped nature of the parameters modification with age. In particular, average speed and kinetic energy were maximal for Older adults and DidRen total time, time from peak acceleration to end of cycle, time to achieve peak acceleration and peak speed were minimal for Older adults.

Table 2 illustrates that Younger and Older adults did not differ significantly for the computed variables. Data’s variation was higher for Children compared to Younger adults for Peak speed and Average speed. For Angle at maximum speed, Seniors and Children showed significantly higher coefficient of variations compared to Younger and Older Adults. Children showed significantly higher coefficient of variations compared to Adults and Seniors for time between peaks acceleration-deceleration. Children showed significantly higher coefficient of variations compared to Adults for time from peak acceleration to end of cycle.

As expected by the distance calculated between the participants and the targets, range of head axial rotation was at approximately 30° with no significant differences between groups for the ROM.

Analyses performed on head-neck complex rotations and trunk rotations were only recorded to verify that trunk motion in the horizontal plane was negligible (0.7±1°).

## Discussion

The objective of the present study was to explore sensorimotor control lifespan performance differences in the dynamics of the head-neck complex. Four age groups including participants from 8 to 85 years performed as fast as possible, 30° amplitude, visually elicited rotations on the right and left sides during a standardized test named DidRen Laser test.

Instructions for the test’s execution were clear for all participants: “you must reach the targets as fast as you can”. In this framework of decision-making, the participants had to trade-off the speed phase and the accuracy phase. As speed-accuracy trade-off in a sensorimotor task such as the DidRen Laser test involved perceptual decisions and cognitive control [41, 42], when instruction emphasizes speed over accuracy, participants tend to be faster and less accurate [41]. Assessment of the speed-accuracy trade-off was not the primary goal of our study, but interestingly it standardized the motion phase. To reduce measurement error, we emphasized maximal effort to standardize the test, and we corrected any subjects’ misunderstanding of what was required during the “warm-up” period. The variability in our results should be seen as reflecting normal variations in the tested participants.

Our participants were asked to focus their attentional resources by aligning the orientations of the eyes and head [43]. This is highlighted by the ROM, which was similar in all age-groups, between (25.5 ± 1.1)° and (26.1 ± 0.8)° [43]. These findings support the fact that the participants respected our theoretical requirement of maximum 30° rotation. Differences in head rotation ROM could have shown a modification in strategies by coupling rotation with another axis of motion and/or trunk rotation to reach the targets. Based on this analysis, we can assume that all studied variables were comparable by being derivates from the axial rotation. Our “one movement dimension test” with head rotation in the horizontal plane met expectation since the test showed appreciable age-related differences performances.

**Fig 6:**
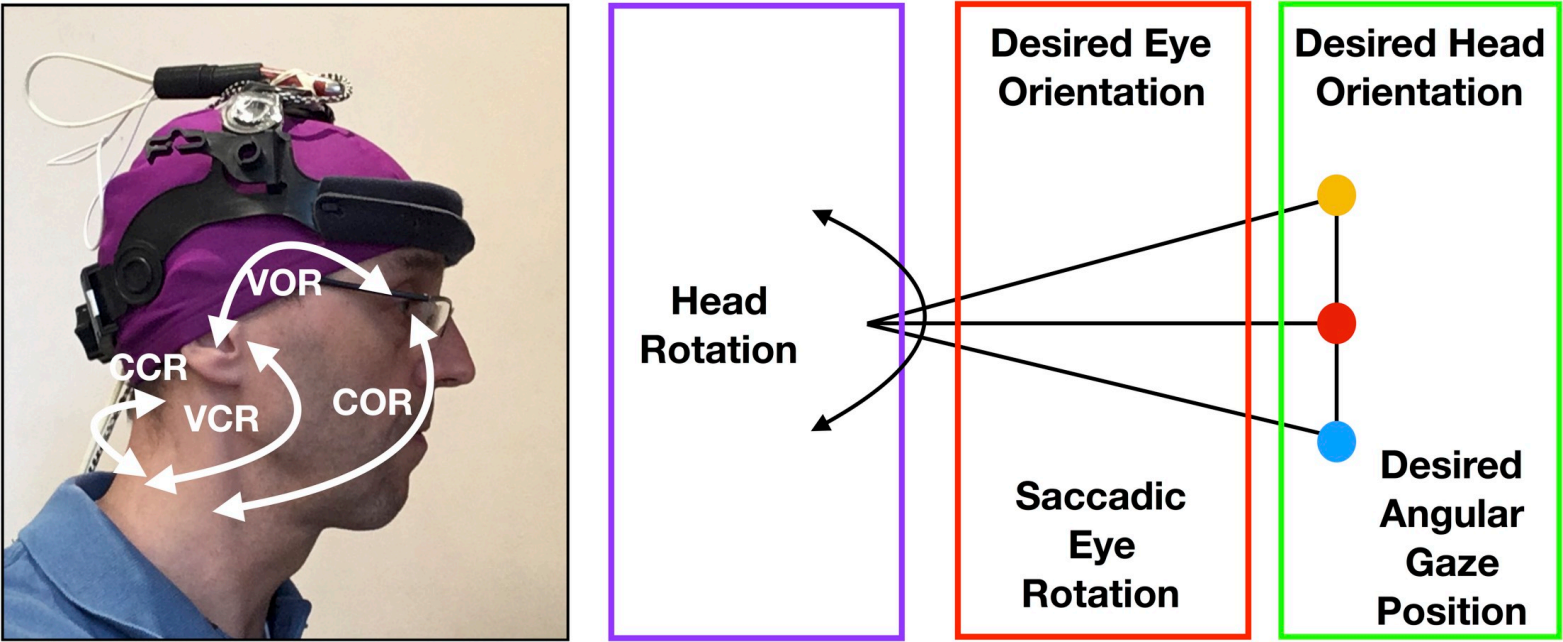
Cervico-collic reflex and flow of information. (A) illustration of reflexes connections associated with cervical afferents: cervico-collic reflex (CCR), cervico-ocular reflex (COR), vestibulo-cervical reflex (VCR) and vestibulo-ocular reflex. (B) flow of information in the DidRen Laser test. Red box shows the part of the model involved in computation of the saccadic eye rotation. Green box shows the part of the model which computes the total head rotation. Violet box shows the VOR predictor related to eye followed by head rotation. Each signal is computed from the signals that have inputs to it.

Sensorimotor control of the head involves patterns of motion that address sensory testing of proprioception, kinesthesis, visuomotor and vestibular control in the plan for meeting a kinematic end [24]. Specific connections exist between the cervical receptors [28, 44], the visual and vestibular systems [24, 28, 45]. This is highlighted by the different reflexes involved with the head, eye and postural stability (Fig 6). The coordination of head and eye movement control stability is ensured by the cervico-collic reflex (CCR), the cervico-ocular reflex (COR) and the tonic neck reflex (TNR) generated by the cervical afferents [22]. The cervico-collic reflex works with the vestibulo-collic reflex (VCR) to activate neck muscles and assist the cervical spine in the maintenance of head position [26]. For rapid head rotation, the vestibular system plays a central role during the saccadic system in coordinating the contributions of the two systems [45]. The cervico-ocular reflex works with the vestibulo-ocular reflexes (VOR) and optokinetic reflexes, which contribute to the calculation of variable timing of eyes saccade and head movement to control the extraocular muscles, creating clear vision during fast head rotation by keeping the eye on target during the latter parts of the head motion [24, 45, 46] (Fig 6).

The results of our study show the presence of age-related differences in rotational sensorimotor control performance of the head-neck complex. Interestingly, no difference was found for all variables studied between Younger (18–35 yrs) and Older adults (36–64 yrs). These results are consistent with those of Bahat et al. (2016) who showed no left and right head rotations difference between three adult groups (18–29 yrs, 30–44 yrs, 45–60 yrs) [16]. However, unlike Bahat et al. (2016) that found significant differences between a group of participants 61–80 yrs and a group of 18–45 yrs during left and right head rotations for peak and mean velocities, we did not observe significant differences between Seniors and other age groups except for DidRen total time (Table 2). This could be explained by our protocol, which emphasized linear head rotation in one plane instead of Bahat’s protocol that considered tridimensional motion for also exploring movements in the other planes.

Two methods assessed the velocity profile. Firstly, the DidRen “target-to-target” reaching task test could be compared to an unconstrained point-to-point hand movement on a horizontal plane trajectory [47, 48]. Participant’s kinematical strategy consists in controlling both his/her instantaneous angular position and speed in view of performing the required task. Therefore, plotting the DidRen typical “target-to-target” motion in a position-speed plane (Fig 4, lower-right panel) is relevant. It shows in a compact form how a given participant manages his/her dynamical degrees of freedom while performing the task. We chose to focus on the angular position at which peak rotational speed was reached. In a “naive” biomechanical picture where the neck would undergo a harmonic motion from 0° to 30° as a torsion spring with equilibrium position 15° would do, the observed bell-shaped profile should be symmetric with respect to an angle of 15°. Such profile was not observed in the present study: the latter angle was lower than 15° for all age groups, hence the acceleration and deceleration phases were managed asymmetrically. This “bell-shaped” profile, which to our knowledge has rarely been investigated in axial cervical rotations [48], thus contains non-trivial dynamical information probably more related to a neuro physiological mechanism explanation. Its structure being independent on the age-groups, it is tempting to conclude that the observed angle-speed profile asymmetry may reflect a well-coordinated healthy motor control. Secondly, the angle-speed diagram does not contain information about motion’s temporal structure which has however a great kinematical interest. For example, relative time to peak velocity has been assessed in straight movements [47–49], and is considered optimal when presenting a temporal symmetry. Along the lines of these studies we assessed time variables such as time from acceleration peak to end of cycle, time between peaks acceleration or deceleration, time to peak speed and the DidRen total time of execution in order to appreciate the participants’ head rotation profile. Only time to peak deceleration and time between peaks of acceleration-deceleration were non-significant. These results contrast with Bahat et al (2016) who reported no difference in velocity profiles among selected age groups using another velocity profile calculation (time to peak velocity percentage), but are in accordance with Flatters et al (2014) who showed that Children exhibited decreased stability in an aiming task [50]. Children took longer time from peak acceleration to end of cycle and reached the peak speed and acceleration significantly later than adults. This observation could seem contradictory except if we consider that Children took more time to achieve peak speed and to perform the test (DidRen total time was longer), suggesting a relationship between these two time variables. According to Leversen et al. (2012), our results demonstrate that Children are less efficient than adults. By referring to Fitts’s law [51], Children are slower than adults to be as accurate as possible [52]. This is consistent with the results of Rothenberg-Cunnigham et al. (2013) who showed age-related limitation in information processing speed [53]: younger Children rely more on spatial end-point and require longer movement time for interception and movement prediction tasks [53]. In summary for the two previous points of discussion, non-temporal and temporal kinematic analyses are complementary approaches to investigate cervical axial fast rotation. Drawing a firm conclusion from these analyses is out of the scope of the present study since it would demand the inclusion of pathological participants in the various age groups.

During head movement, the development of muscle forces with a specific timing activity [54] are required to generate rotational kinetic energy necessary to move the head-neck complex. This kinetic energy depends on the moment of inertia and the angular speed of the head. To our knowledge, no study assessed the head’s moment of inertia in Children neither estimated the amount of energy required to perform such rotational motion. We calculated it for Children and Adults (females/males) and used it for rotational kinetic energy computation [39, 40]. The average rotationnal kinetic energy is proportional to $m\;R^{2}\;\overset{-}{\omega^{2}}$, with *m* and *R* the head mass and radius and $\overset{-}{\omega^{2}}$ the average squared angular speed. The parameters *m* and *R* are obviously smaller in children, and our analysis showed that the average angular speed was lower for children. So, it is not surprising that Children developed less kinetic energy compared to other age-groups. Age-related kinetic differences can therefore be explained by anthropometric proportions (head’s moment of inertia) and also by neuromuscular immaturity (lower angular speed).

Previous clinical studies including neck pain patients [48, 49, 55] showed that head-neck complex rotational movements and their derivatives could bring relevant insight on sensorimotor control, and our results go along these lines. The variables assessed in this study do not distinguish between specific sensorimotor subsystems [29], but it is conceivable that Children-related performances may be attributable to slow sensorimotor system maturation [17, 56] and central nervous system development immaturity [4, 17, 56–58]. Lifespan development showed a linear reduction of performance in tasks that were dependent on speed [4]. Indeed, aging modifications [10, 16] can be attributed to dysfunction in neck proprioception, vestibular and visual systems [10, 11]. But, to our surprise, in our study, the senior age-related group did not show differences with kinematic variables computed from speed. It means that if Seniors did not show significant difference for kinematic variables (during the rotation phase) the difference highlighted by the total test time (only the total time allowed to discriminate Seniors from Younger adults) could be attributable to the accuracy phase. Lafargue et al. (2013) showed that inadequate updating of physical capabilities with aging could be viewed as a combination of impaired updating and decreased physical ability, which may lead to over-optimistic predictions about the feasibility of actions. In other words, the fact that Seniors behaved in much the same way only in head rotation phase as adults’ participants suggests that motor imagery capacity is relatively unaffected by age. Hence, we believe that the DidRen total time is the most relevant outcome, because it takes in consideration the two phases of the test (motion and stabilization). In view of foregoing, it emerges that our results go along previous clinical studies including patients with neck pain [48, 49, 55], which showed that head-neck complex rotational movements and their derivatives could bring relevant insight on sensorimotor control.

Data dispersion around the average values, i.e. in the CV, could be indicative of an important source of variability in Children movements (see also Fig 5). As discussed before, testing adequacy could be determined by the increasing probability of error when movement speed increases. Thus, our results support the hypothesis that Children, in a different way compared to adults and sometimes Seniors, integrate both the inherent motor variability and the cost of failure when planning and controlling fast target aiming movements like the DidRen Laser test [59]. Therefore, to compensate for increased inherent motor variability, Children reduced average speed (Table 2) appropriately in order to reduce the probability of missing the target. Speed and derived kinematic variables, which are maximal in the adult groups, while variability is minimal, could indicate that the latter age-group reaches an optimal efficiency in the performed motion. There appeared to be a close connection between the relative kinematic variability of the Children data and the relative performance quantity. Average speed showed significant differences in terms of quantity (see Average speed results) and showed quality significant difference between age-groups: Children showed more variation when compared to Young adults. Thus, the modest explanation is that variability could be the main driver [59].

U-shaped or inverted U-shaped curve age profile emphasizes differences related to aging [4, 5]. The graphical representations show gradual increase of performance from Children to Younger and Older adults, and decrease of performance from Younger and Older adults to Seniors (Fig 5). This finding could be interpreted, according to Leversen et al. (2012), as a general pattern of life-span development [4, 15].

This study has some limitations. Many outcomes were investigated, and it could be viewed in term of limitation, but our goal was to explore in-depth all possible kinematic variables without preexisting thinking. Calculation of the trunk rotations on 5 participants of each age-group could be seen as limitation but our goal was to simplify the set up in order to use, in the future, only one inertial measurement unit placed on the head. Moreover, axial head rotation relative to the trunk was not calculated because we wanted to be more functional without given instruction to keep the trunk steady. We could have assessed sagittal plane motion since extension, according to Saavedra et al. (2012), is significantly associated with self-reported disability in chronic mechanical neck pain [60]. However, we chose to study motion in the horizontal plane since rotation is considered the most usual head-neck complex motion performed during daily life activities [61]. Our current estimates of the rotational kinetic energies do not allow us to go beyond obvious anthropometric-induced differences between Children and Adults. A simultaneous measure of muscular activity may help to correlate energetical variations to age-related changes of muscular activation patterns. We leave such an issue for future works.

The use of cross-sectional design can be considered as limitation and in the future longitudinal studies would be valuable. Small women and men sample size did not allow us to analyze gender influence and that needs to be addressed in future work.

Our data of asymptomatic participants across the lifespan provide important information for physiologists, biomechanicians and clinicians assessing patients with neck pain and or dysfunction, allowing comparisons and qualitative and quantitative targets of rehabilitation for head-neck rotation motor control tasks.
